# PD1-CD28 Fusion Protein Enables CD4+ T Cell Help for Adoptive T Cell Therapy in Models of Pancreatic Cancer and Non-hodgkin Lymphoma

**DOI:** 10.3389/fimmu.2018.01955

**Published:** 2018-08-30

**Authors:** Felicitas Rataj, Fabian B. T. Kraus, Michael Chaloupka, Simon Grassmann, Constanze Heise, Bruno L. Cadilha, Peter Duewell, Stefan Endres, Sebastian Kobold

**Affiliations:** ^1^Center of Integrated Protein Science Munich (CIPS-M) and Division of Clinical Pharmacology, Department of Medicine IV, Klinikum der Universität München, Member of the German Center for Lung Research (DZL), Munich, Germany; ^2^German Cancer Research Center (DKTK), Partner Site Munich, Heidelberg, Germany

**Keywords:** adoptive T cell transfer, cancer immunotherapy, costimulation, PD-1-CD28 fusion protein, CD4+ T cells

## Abstract

**Background:** Interaction of the programmed death receptor 1 (PD-1) and its ligand, PD-L1, suppresses T cell activity and permits tumors to evade T cell-mediated immune surveillance. We have recently demonstrated that antigen-specific CD8+ T cells transduced with a PD1-CD28 fusion protein are protected from PD-1-mediated inhibition. We have now investigated the potential of PD1-CD28 fusion protein-transduced CD4+ T cells alone or in combination with CD8+ T cells for immunotherapy of pancreatic cancer and non-Hodgkin lymphoma.

**Methods:** OVA-specific CD4+ and CD8+ were retrovirally transduced with the PD1-CD28 fusion protein. Cytokine release, proliferation, cytotoxic activity, and phenotype of transduced T cells were assessed in the context of Panc02-OVA (murine pancreatic cancer model) and E.G7-PD-L1 (murine T cell lymphoma model) cells.

**Results:** Stimulation of PD1-CD28 fusion protein-transduced CD4+ T cells with anti-CD3 and recombinant PD-L1 induced specific T cell activation, as measured by IFN-y release and T cell proliferation. Coculture with Panc02-OVA or E.G7-PD-L1 tumor cells also led to specific activation of CD4+ T cells. Cytokine release and T cell proliferation was most effective when tumor cells simultaneously encountered genetically engineered CD4+ and CD8+ T cells. Synergy between both cell populations was also observed for specific tumor cell lysis. T cell cytotoxicity was mediated via granzyme B release and mediated enhanced tumor control *in vivo*. Transduced CD4+ and CD8+ T cells in co-culture with tumor cells developed a predominant central memory phenotype over time. Different ratios of CD4+ and CD8+ transduced T cells led to a significant increase of IFN-y and IL-2 secretion positively correlating with CD4+ T cell numbers used. Mechanistically, IL-2 and MHC-I were central to the synergistic activity of CD4+ and CD8+ T cells, since neutralization of IL-2 prevented the crosstalk between these cell populations.

**Conclusion:** PD1-CD28 fusion protein-transduced CD4+ T cells significantly improved anti-tumoral effect of fusion protein-transduced CD8+ T cells. Thus, our results indicate that PD1-CD28 fusion protein-transduced CD4+ T cells have the potential to overcome the PD-1-PD-L1 immunosuppressive axis in pancreatic cancer and non-Hodgkin lymphoma.

## Introduction

Cytotoxic T cells specifically recognize tumor antigens presented on major histocompatibility complex-1 (MHC-I). After binding to the tumor antigen in the context of MHC, T cells are activated, which results in the secretion of cytotoxic factors and target cell lysis (1, 2). This concept is utilized therapeutically for adoptive T cell therapy (ACT). Patient-derived, tumor-specific T cells are expanded *ex vivo* or, to further enhance tumor-specificity, are genetically modified. T cell engineering usually follows two main approaches; either by introducing a T cell receptor specific for a given tumor-associated antigen or by equipping T cells with chimeric antigen receptors (CAR), which are synthetic receptors enabling tumor recognition. Following expansion, T cells are infused back to the patient in therapeutic intention (3). Pioneering work for ACT utilized tumor-infiltrating lymphocytes (TIL) for melanoma treatment yielding consistent durable response rates in subsets of patients. The challenges to generate these cells from tumor tissue of individual patients or even across entities has so far refrained this strategy from large scale clinical testing (4). Based on compelling preclinical and clinical data in hematological malignancies, ACT holds great promise for cancer immunotherapy. In 2017, the Food and Drug Administration (FDA) approved the first cellular therapy for refractory B-cell acute lymphoblastic leukemia (B-ALL) and diffuse large B cell lymphoma. Anti-CD19-CAR T cells are now part of the standard of care in the US, based on unparalleled remission rates and prolonged overall survival for patients with an otherwise very poor prognosis (5). In addition, ACT is under investigation for the treatment of other hematologic as well as more frequent non-hematological malignancies. Typically, ACT is performed with a mixture of CD4+ and CD8+ T cells, which is dictated by the patient's own peripheral blood T cell ratio and the differential expansion status in cell culture. Some protocols also adjust for defined ratios, based on own evidence that this might be more beneficial (6–8). When being transduced for tumor specificity both cell types are being modified and in the case of CAR T cells, both cell populations are thought to be therapeutically relevant (9).

However, CD8+ T cells are generally considered more potent and more central for ACT efficacy. CD4+ T cells have a distinct functional and secretory phenotype from CD8+ T cells which is neither redundant nor overlapping. Importantly, CD4+ T cell-derived cytokines play an important role in anti- but also in pro-tumoral immunity (10, 11). While it is established that CD4+ T cells can be cytotoxic on their own, a major function lays in regulating trafficking, activation, proliferation, differentiation, and persistence of tumor-infiltrating cytotoxic CD8+ T cells (12–15). Several studies have confirmed the helper function of tumor-specific CD4+ T cells and showed that the anti-tumor activity of combined treatment with CD4+ and CD8+ T cells is more pronounced than that seen when using individual cell types. The exact mechanism of this synergy remains to be elucidated (16–18).

Despite the clinical success of ACT in defined indications, ACT is inherently limited by antigen-loss variants of tumor cells, side effects resulting from on- and off-target expression of the chosen antigen and low T cell infiltration into the tumor tissue. ACT failure is often associated with an increased expression of the programmed death-1 receptor (PD-1), a marker protein for T cell anergy, on previously activated T cells (19, 20). PD-1 signaling mediates T cell suppression that prevents autoimmunity under physiological conditions and is therefore a key immune checkpoint on CD4+ and CD8+ T cells (21, 22). PD-L1, one of the two known ligands for PD-1, is broadly expressed on epithelial as well as hematological cells and shields these cells from T cell overactivation (23). Along these lines, tumors usurp this mechanism to evade anti-tumor immune responses (24). It is thereby not surprising, that undulating PD-L1 expression is found in most if not all human cancers at different levels and its expression is associated with dismal prognosis in the pre-immunotherapy era (25). Paradoxically, recognition of tumor cells by T cells transferred for ACT will result in T cell activation, upregulation of PD-1 on the said T cell, but also of PD-L1 on the tumor cell. This will ultimately end in abrogation of T cell activity and thereby ACT failure (26). Clinical evidence that this state of anergy might be reverted when antagonizing the PD-1-PD-L1 axis has been shown in several phase III clinical trials testing anti-PD-1 or anti-PD-L1 antibodies in melanoma or non-small cell lung cancer (27–31). Based on these studies, it seems likely that a similar approach might also be of value for ACT. As both checkpoint blockade and ACT have severe side effects on their own, it might be advisable to develop more targeted strategies to overcome T cell anergy than systemically blocking important immune checkpoints.

To overcome PD-1 suppression selectively and to improve ACT, we have developed a therapeutic concept that converts tumor-associated immunosuppression via the PD-1-PD-L1 axis into stimulation of tumor-specific T cells (32). We created a fusion receptor consisting of the extracellular domain of the PD-1 receptor fused to the intracellular, T cell-activating domain of CD28. In the tumor tissue, PD-1-CD28 fusion protein-expressing CD8+ T cells recognize tumor-derived PD-L1 and get locally activated. This results in tumor cell lysis and therapeutic benefit. It, however, remained unclear if the benefit is specific to CD8+ T cells, and particularly if adding this fusion protein to CD4+ T cells would further accelerate therapeutic activity. We hypothesized that our PD-1-CD28 fusion protein is not only functional in antigen-specific CD4+ T cells but also that simultaneous introduction in CD8+ T cells would further enhance T cell function. Here, we demonstrate that primary murine CD4+ T cells, expressing PD1-CD28 fusion protein, overcome PD-L1-induced T cell anergy in murine models of pancreatic cancer and non-Hodgkin lymphoma. Coculture experiments demonstrate a synergism of gene-modified CD4+ and CD8+ T cells for anti-tumor activity, which was dependent on IL-2 secretion from CD4+ T cells. Our results indicate the potential of PD1-CD28 fusion protein-transduced CD4+ T cells to further improve ACT.

## Materials and methods

### Cell lines

Panc02-OVA, a murine pancreatic cancer cell line and E.G7-OVA, a murine T cell lymphoma cell line, were previously described (33, 32). Panc02-OVA-PD-L1 and E.G7-OVA-PD-L1 were generated by transduction of Panc02-OVA or E.G7-OVA cells with pMXs-puro or pMXs (a generous gift from Toshio Kitamura, M.D., PhD, the Institute of Medical Science, University of Tokyo, Japan) encoding the full-length murine PD-L1 (Swiss-Prot accession number Q9EP73). Panc02-OVA-PD-L1 cells were selected based on puromycin resistance. E.G7-OVA-PD-L1 cells were obtained by fluorescence activated cell sorting. Panc02-OVA and Panc02-OVA-PD-L1 were cultured in DMEM3+ (DMEM with 10 % fetal bovine serum [FBS, Life Technologies, USA), 100 U/ml penicillin and streptomycin (PS), and 2 mM L-glutamine (all from PAA, Germany)]. E.G7-OVA-PD-L1 were cultured in RPMI 1,640 supplemented with 10% FBS, 100 U/ml PS and 2 mM L-glutamine, 1 mM sodium pyruvate (PAA, Germany), and 1 mM HEPES (Sigma Aldrich, Germany).The retroviral ecotrophic packaging cell line Platinum-E was purchased from Cell biolabs (USA). DMEM3+ medium for Platinum-E cells additionally contained 10 μg/ml puromycin and 1 μg/ml blasticidin (both from Sigma, Germany). Primary murine T cells were cultured in RPMI 1640 supplemented with 10% FBS, 100 U/ml PS and 2 mM L-glutamine, 1 mM sodium pyruvate (PAA, Germany), 1 mM HEPES (Sigma Aldrich, Germany), and 50 μM β-mercaptoethanol.

### Mice

Mice transgenic for a T cell receptor specific for ovalbumine (OT-1 or OT-2) were obtained from the Jackson laboratory (Bar Harbor, ME) (stock number 003831 for OT-1 and 004194 for OT-2) and were bred in our animal facility under SPF conditions. Both mouse strains served as T cell donors for primary murine T cell transduction.

### Animal experiments

For *in vivo* studies wild type C57/Bl6 mice were purchased from Charles River. Tumors were induced by subcutaneous injection of 4 x 10^5^ E-G7-OVA-PD-L1 tumor cells. Mice were randomized with regard to tumor size and treated via serial transfer of PTM-transduced or untransduced T cells: First, CD8+ T cells were injected i.v. 48 h later, CD4+ T cells were injected i.v. Tumor growth was assessed every other day in a blinded fashion and tumor volume was estimated according to the following formula: 4/3 x π x ${\text{L}}_{1}^{2}$ x L_2_ (with L_1_ defined as maximal diameter and L_2_ as the diameter perpendicular to L_1_). All experiments were approved by the local regulatory agency (Regierung von Oberbayern).

### T cell transduction

The PD1-CD28 fusion protein was described previously (32). The retroviral vector pMP71 (kindly provided by Christopher Baum, M.D., Institute of Experimental Hematology, Medizinische Hochschule Hannover, Germany) was utilized for all transduction experiments. Detailed protocols for murine T cell transduction have been published (34–37). In brief, pMP71 PD1-CD28 vector was transfected into Platinum-E cells and retrovirus-containing supernatants were collected for transduction of murine T cells. Primary murine T cells were first stimulated with anti-CD3e and anti-CD28 antibody (eBioscience, clones 145-2C11 and 37.51, respectively) and recombinant IL-2 (Novartis, Switzerland). Priot to transduction, anti-CD3- and anti-CD28 beads (Life technologies, USA) were added. Recombinant IL-15 (Peprotech, Germany) was used for T cell expansion. The CD4+ T cell fraction was purified on the day of spleen extraction by magnetic activated cell sorting using a CD4+ T cell isolation kit (Miltenyi Biotec, Germany).

### Flow cytometry

For multi-color flow cytometry, a BD FACS Canto II (BD bioscience, Germany) together with the following antibody panels was used. For purity testing and analysis of transduction efficiencies, anti-PD-1 (APC, clone RMP-30, BioLegend, USA), anti-CD8 (Pacific Blue^TM^, clone 53-6.7, BioLegend, USA) and anti-CD4 (Pacific Blue^TM^ e, clone GK1.5, BioLegend, USA) were used. For analysis of MHC I-, MHCII-, and PD-L1-expression, tumor cells were stained with anti-MHCI (PE, clone M1/42.3.9.8, Elabscience, USA), anti-MHCII (APC, clone M5/114.15.2, eBioscience, USA) and anti-CD274 (PE/Cy7, clone 10F.9G2, BioLegend, USA). Rat IgG2a– (PE, clone #54447, R&D Systems, USA), Rat IgG2b kappa—(APC, clone eB149/10H5, eBioscience, USA) and Rat IgG2b kappa—antibodies (PE/Cy7, clone RTK4530, BioLegend, USA) were applied as isotype control. For proliferation analysis in antibody-stimulation assays, T cells were stained with anti-PD-1 (APC, clone RMP-30, BioLegend, USA), anti-CD28 (APC, clone 37.51, BioLegend, USA), anti-CD4 (Pacific Blue^TM^, clone GK1.5, BioLegend, USA), and Zombie aqua fixable viability dye (BioLegend, USA) prior to fixation and permeabilization with FoxP3/Transcription Factor Staining Buffer Set (eBioscience, USA). For staining of intracellular proteins, anti-Ki67 (PE, clone 16A8, BioLegend, USA) and anti-EOMES (PE/Cy7, clone DAN11mag, eBioscience, USA) were added. Cells were washed and resuspended in PBS (Lonza, Switzerland) containing count bright absolute counting beads (Life technologies, USA). For proliferation analysis in cocultures of T cells and tumor cells, T cells were stained with anti-PD-1 (APC, clone RMP-30, BioLegend, USA), anti-CD4 (Pacific Blue^TM^, clone GK1.5, BioLegend, USA), anti-CD8 (APC/Cy7, clone 53-6.7, BioLegend, USA), and Zombie aqua fixable viability dye (BioLegend, USA). Equal amounts of counting beads (Life technologies, USA) were added to each sample. The antibody panel for T cell phenotyping consisted of anti-PD-1 (FITC, clone 29F.1a12, BioLegend, USA), anti-CD8 (APC/Cy7, clone 53-6.7, BioLegend, USA), anti-CD4 (PE/Cy7, clone RM4-5, BioLegend, USA), anti-CD62L (Pacific Blue^TM^, clone MEL-14, BioLegend, USA), anti-CCR7 (PerCP/Cy5.5, clone 4B12, BioLegend, USA), and Zombie aqua fixable viability dye (BioLegend, USA).

### MHC I-, MHC II-, and PD-L1-profiling of tumor cells

For the analysis of MHC I-, MHC II-, and PD-L1-expression on Panc02-OVA and E.G7-OVA-PD-L1, 5 × 10^4^ tumor cells were stimulated for 48 h with recombinant murine IFN-γ (Peprotech, USA) at increasing concentrations of 2, 20, or 100 ng/ml respectively and analyzed by flow cytometry as described above.

### Antibody-stimulation assays

For antibody-stimulation assays, T cells were stimulated with anti-CD3 antibody (100 ng/ml, clone 145-2C11, eBioscience), anti-CD3 antibody and recombinant PD-L1-Fc chimera protein (5 μg/ml, R&D Systems) or anti-CD3 antibody and anti-CD28 antibody (2 μg/ml, clone 37.51, eBioscience) for 48 h. Mitotic activity and CD28 surface expression was analyzed by flow cytometry. Cells were stained as indicated and cell numbers were normalized with counting beads (Life Technologies, Germany). Cytokine release was quantified by ELISA (IL-2 and IFN-γ, both BD).

### Cocultures of T cells and tumor cells

For T cell-tumor cell cocultures, CD8+ and CD4+ T cells (in a 3:1, 1:1, or 1:3 cell ratio) were prestimulated with anti-CD3 antibody (100 ng/ml, clone 145-2C11, eBioscience) and recombinant PD-L1-Fc chimera protein (5 μg/ml, R&D Systems) for 24 h, as described above. T cells were then cocultured for 16 h with either E.G7-OVA-PD-L1, Panc02-OVA, or Panc-OVA-PD-L1 tumor cells in a 10:1 effector to target cell ratio. Cytokine release was quantified by ELISA (IL-2 and IFN-γ). For cytotoxicity assays, tumor cell-derived LDH release was quantified after 16 h using CytoTox 96® Non-Radioactive Cytotoxicity Assay (Promega, USA). Granzyme B secretion was determined using Mouse Granzyme B DuoSet® ELISA (R&D systems, USA). For T cell phenotyping and proliferation assays, T cells were cocultured with Panc-OVA-PD-L1 for 36 h, as described above. T cell phenotype and proliferation was analyzed by flow cytometry as described above.

### MHC I, MHC II, and IL-2 neutralization assays

For MHC I, MHC II, and IL-2 neutralization experiments, CD8+ and CD4+ T cells (in a 1:1 ratio) were prestimulated with anti-CD3 antibody and recombinant PD-L1-Fc chimera for 24 h. Subsequently, T cells and Panc02-OVA cells were cocultured at a 10:1 effector to target cell ratio. Anti-mouse MHC class I antibody (10 μg/ml, clone M1/42.3.9.8, *InVivo*MAb), anti-mouse MHC class II antibody (10 μg/ml, clone M5/114.15.2, eBioscience) and LEAF purified anti-mouse IL-2 antibody (10 μg/ml, clone JES6-1A12, BioLegend) were added during prestimulation and co-culture. Supernatants were analyzed for IFN-γ by ELISA.

### Statistical analysis

For statistical analysis, GraphPad Prism software version 7.04 was used. Reported values are continuous. Differences between experimental conditions were analyzed using the unpaired two-sided Student's *t*-test. *P*-values < 0.05 were considered as significant. Data shown are mean values ± SEM of at least three biological replicates representative for three independent experiments as indicated.

## Results

### Functional analysis of PD1-CD28 fusion protein (PTM) in CD4+ T cells

**Figure 1:**
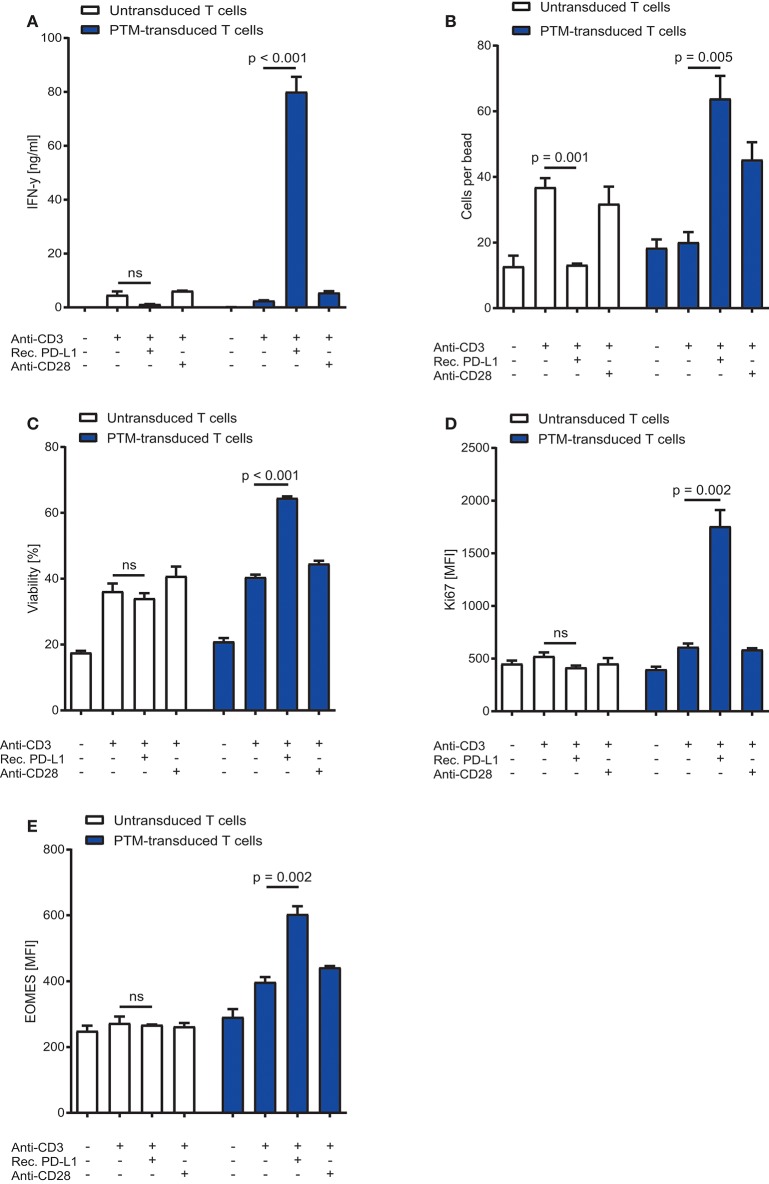
*In vitro* characterization of PD1-CD28 fusion protein (PTM)-transduced CD4+ T cells. PTM-transduced or untransduced primary murine CD4+ T cells were either stimulated with anti-CD3 antibody, anti-CD3 antibody, and recombinant PD-L1 or anti-CD3 antibody and anti-CD28 antibody. **(A)** Interferon-γ (IFN-γ) secretion was measured by enzyme linked immunosorbent assay (ELISA). **(B)** T cell number was analyzed by flow cytometry and normalized to standardized counting beads. **(C)** Viability of T cells was assessed by flow cytometry. **(D)** After 48 h of stimulation T cells were intracellularly stained for Ki67, a mitosis marker or **(E)** for the differentiation marker eomesodermin (EOMES). Experiments **(A–E)** are representative of three independent experiments each performed in triplicates. Bars represent SEM and *P* values from Student's *t*-test are shown. All tests are two-sided.

To characterize the functionality of PTM in CD4+ T cells, we transduced PTM into primary murine CD4+ T cells. PTM-transduced and untransduced T cells were then stimulated with anti-CD3 antibody, anti-CD3 antibody and recombinant PD-L1 or anti-CD3 antibody and anti-CD28 antibody for 48 h. CD4+ PTM-transduced T cells showed significantly higher IFN-γ release as compared to untransduced T cells (Figure 1A). T cell activation was paralleled by an increase in T cell viability and T cell proliferation (Figures 1B,C). Untransduced CD4+ T cells were more strongly stimulated by anti-CD3 than PTM-transduced CD4+ T cells, while combination with anti-CD28 antibodies brought PTM-transduced T cells to a similar level of stimulation as untransduced T cells in this control condition. Similarly, expression of the mitogenic marker Ki67 was higher in PTM-transduced T cells than in untransduced T cells (Figure 1D). Expression of Eomesodermin (EOMES), a T cell differentiation marker, was highest for anti-CD3 and PD-L1-stimulated, transduced T cells compared to untransduced cells (Figure 1E). Together, these results demonstrate that PTM is functional in CD4+ T cells and enhances their functionality.

### Functional analysis of PTM-transduced T cells cocultured with tumor cells

**Figure 2:**
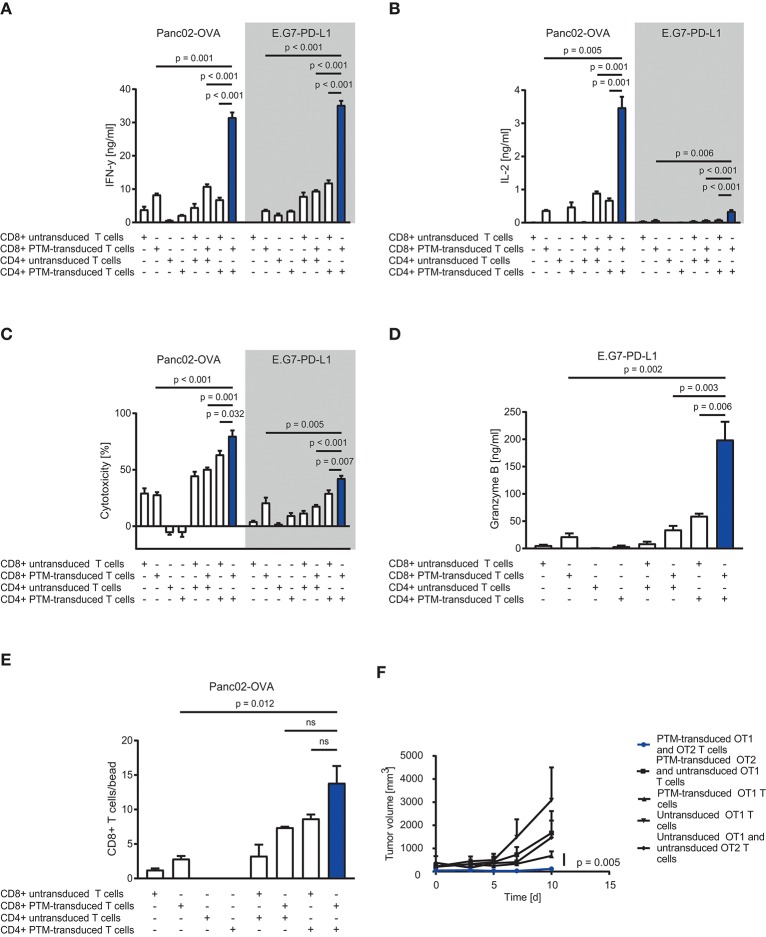
*In vitro* and *in vivo* assessment of anti-tumor efficacy of PD1-CD28 fusion receptor (PTM receptor)-transduced CD4+ and CD8+ T cells. **(A)** PTM-transduced, untransduced primary murine OT-1, PTM-transduced, untransduced primary murine OT-2 T cells, or OT-1 together with OT-2 T cells were prestimulated for 24 h with anti-CD3 antibody plus recombinant PD-L1. T cells were then cocultured with Panc02-OVA or E.G7-PD-L1 cells. Interferon-γ (IFN-γ) secretion was measured by enzyme linked immunosorbent assay (ELISA). **(B)** Interleukin-2 (IL-2) release was measured by ELISA. **(C)** PTM-transduced, untransduced primary murine OT-1, PTM-transduced, untransduced primary murine OT-2 T cells, or OT-1 together with OT-2 T cells were prestimulated for 24 h with anti-CD3 antibody and recombinant PD-L1. In the meantime, Panc02-OVA or E.G7-PD-L1 cells were seeded and grown prior to the addition of T cells. LDH release measurement from lysed tumor cells was performed after 16 h of coculture. **(D)** Granzyme B secretion by T cells cocultured with E.G7-PD-L1 cells for 16 h measured by ELISA. **(E)** PTM-transduced, untransduced primary murine OT-1, PTM-transduced, untransduced primary murine OT-2 T cells or OT-1 together with OT-2 T cells were prestimulated for 24 h with anti-CD3 antibody plus recombinant PD-L1 and then cocultured with Panc02-OVA cells. T cell numbers were analyzed by flow cytometry and normalized to standardized counting beads. **(F)** 30 mice were subcutaneously injected with E.G7-OVA-PD-L1 tumor cells in two independent experiments. As soon as all tumors were established, the mice were randomized, assigned to five different treatment groups and treated with either PTM-transduced (*n* = 6) or untransduced primary murine OT1 T cells (*n* = 7) or with PTM-transduced (*n* = 4) or untransduced (*n* = 4) primary OT2 T cells in combination with OT1 T cells or PTM-transduced OT-1 T cells (*n* = 9). Tumor growth was assessed every other day in a blinded fashion and tumor volume was calculated as indicated. Pooled data from two independent experiments is shown here. Curves are censored by the time the first mice had to be taken out of the experiment either due to tumor size or ulceration (day 10). Experiments **(A–E)** are representative of three independent experiments each performed in triplicates. Experiment **(F)** represents pooled data of two independent experiments. Bars represent SEM and *P* values from Student's *t*-test are shown. All tests are two-sided.

To assess the therapeutic potential of PTM-transduced CD4+ T cells *in vitro*, we prestimulated antigen-specific CD4+ or CD8+ PTM-transduced or untransduced T cells at a ratio of 1:1 with anti-CD3 antibody and recombinant PD-L1 for 24 h. Prestimulation was performed to mimic primary antigen contact and to induce partial anergy of the cells, as expected in the tumor environment. CD4+ or CD8+, untransduced, or transduced T cells were then cocultured alone or in different combinations with either Panc02-OVA cells or E.G7-PD-L1 cells. PTM-transduced CD4+ and CD8+ T cells produced more IFN-γ in contact with either cell line compared to untransduced T cells (Figure 2A). Highest IFN-γ secretion was measured for both tumor cell lines when PTM-transduced CD4+ and PTM-transduced CD8+ were combined. The same effect was observed for IL-2 release (Figure 2B). T cell activation was followed by a similar effect on T cell-mediated cytotoxicity. CD4+ and CD8+ PTM-transduced T cells, prestimulated individually, induced significant lysis of Panc02-OVA and E.G7-PD-L1 cells as compared to untransduced T cells (Figure 2C). Similar to cytokine production, cytotoxic activity was highest, when CD4+ and CD8+ PTM-transduced T cells were cocultured with tumor cells as compared to control conditions. Mechanistically, T cell cytotoxicity correlated with granzyme B release indicating that T cell degranulation is the mode of action, which is boosted by PTM transduction (Figure 2D). T cell cytotoxicity was accompanied by an increase in the number of CD8+ T cells in coculture with CD4+ T cells and Panc02-OVA (Figure 2E). PTM-transduced CD4+ T cells in coculture with Panc02-OVA-PD-L1 cells developed a predominant central memory phenotype, defined by CCR7+ and CD62L+ expression, over time (Supplementary Figure 1A). The effect on CD4+ T cells was strongest in the presence of untransduced or PTM-transduced CD8+ T cells. However, PTM-expression on CD8+ T cells alone, did not have an influence on the CD4+ T cell phenotype. CD8+ T cells, in contrast, differentiated into central memory T cells within the same experimental setting (Supplementary Figure 1B). In these cocultures, the amount of effector memory T cells was reduced in both, CD4+ and CD8+ T cells transduced with PTM (Supplementary Figures 1C,D). Our results suggest that CD4+ PTM-transduced T cells have therapeutic activity *in vitro* and point toward a synergistic collaboration of CD4+ and CD8+ T cells. Of note, this effect was highest when PTM was expressed by both T cell subsets. *In vivo*, combined treatment of OT1-PTM with OT2-PTM T cells mediated enhanced tumor control over PTM-transduced OT1 T cells, OT1 plus OT2 T cells and OT1 plus PTM-OT2 T cells in the EG7-PD-L1 model (Figure 2F). These results indicate the potential value of the strategy *in vivo*.

### CD4+ to CD8+ T cell ratio positively influence the activity of PTM-transduced T cells via IL-2 in coculture with tumor cells

**Figure 3:**
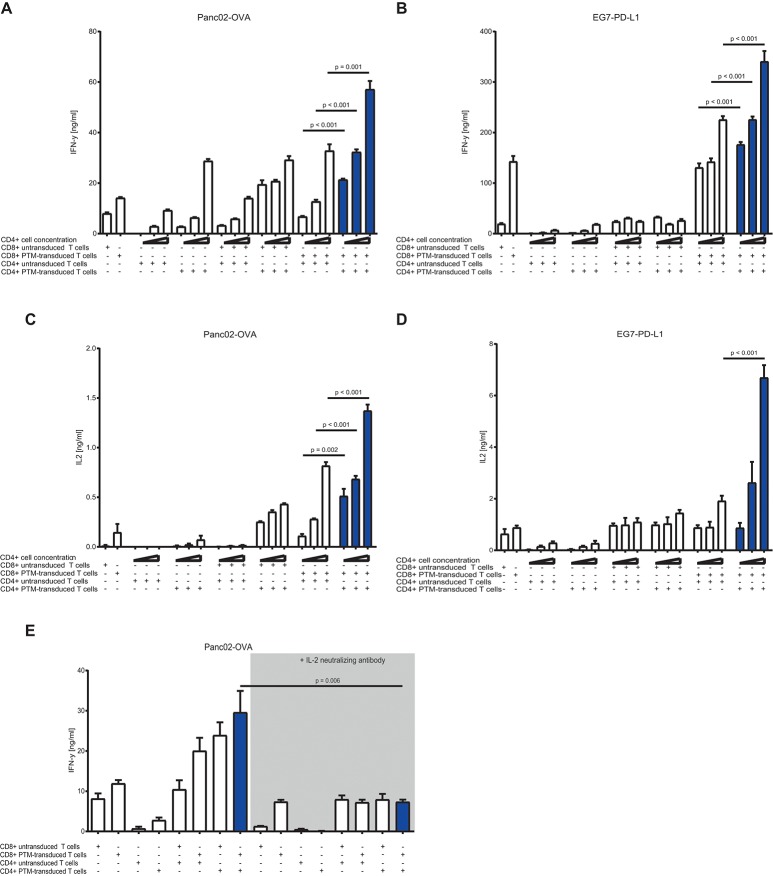
*In vitro* characterization of PD1-CD28 fusion protein (PTM)-transduced CD4+ and CD8+ T cells in T cell-tumor cell cocultures at different CD4+ to CD8+ T cell ratios in the presence or absence of a neutralizing anti-IL-2-antibody. **(A,B)** PTM-transduced or untransduced primary murine OT-1, PTM-transduced or untransduced primary murine OT-2 T cells or combinations of these were prestimulated for 24 h with anti-CD3 antibodies plus recombinant PD-L1. Three different ratios of CD4+ to CD8+ T cells were applied (i.e., 3:1, 1:1, or 1:3 CD4+ to CD8+ T cell ratio). After prestimulation, the T cells were cocultured with Panc02-OVA or E.G7-PD-L1 cells for a further 48 h. The resulting Interferon-γ (IFN-γ) release was measured by enzyme linked immunosorbent assay (ELISA). **(C,D)** The concentration of interleukin-2 (IL-2) in the supernatants was measured by ELISA. **(E)** PTM-transduced or untransduced primary murine OT-1, PTM-transduced or untransduced primary murine OT-2 T cells or combinations of these were prestimulated for 24 h with anti-CD3 antibodies plus recombinant PD-L1. T cells were then cocultured with Panc02-OVA. In the blocking condition, a neutralizing anti-IL-2 antibody was present during the period of prestimulation and coculture. The resulting IFN-γ release was measured by ELISA. Experiments **(A–E)** are representative of three independent experiments each performed at least in triplicates. Bars represent SEM and *P* values from Student's *t*-test are shown. All tests are two-sided.

To test the CD4+ to CD8+ T cell ratio with the highest synergistic potential, we prestimulated antigen-specific, untransduced, or PTM-transduced CD8+ T cells and increasing numbers of antigen-specific, untransduced, or PTM-transduced CD4+ T cells with anti-CD3 antibody plus recombinant PD-L1. CD4+- or CD8+-, untransduced or transduced T cells were then cocultured alone or in different combinations with either Panc02-OVA or E.G7-PD-L1. In both tumor models, IFN-γ secretion, as indicator for T cell activation, was highest when PTM+ CD4+ and PTM+ CD8+ T cells were combined (Figures 3A,B). IFN-γ level positively correlated with the number of CD4+ T cells present in the coculture, accompanied by comparable IL-2 levels (Figures 3C,D). IL-2 levels were highest when PTM+ CD4+ and PTM+ CD8+ were cocultured with target cells. IL-2 production was tightly correlated with the number of CD4+ cells, pointing toward a potential role of IL-2 in their collaborative activity. To test this hypothesis, T cells were prestimulated and incubated with Panc02-OVA cells in the presence of anti-IL-2 neutralizing antibody. T cell activation, measured by IFN-y release, was almost abrogated through neutralization of IL-2 (Figure 3E). Similarly, synergy in T cell cytotoxicity was also blocked by anti-IL-2 neutralizing antibody in cocultures of Panc02-OVA cells with PTM+ CD4+ and PTM+ CD8+ T cells (Supplementary Figure 2). Taken together, our results demonstrate that the synergistic effect of transduced CD4+ and CD8+ T cells is dose-dependent and is mediated by IL-2.

### Synergistic activity is dependent on PD-L1 and MHC I but not on MHC II expression

**Figure 4:**
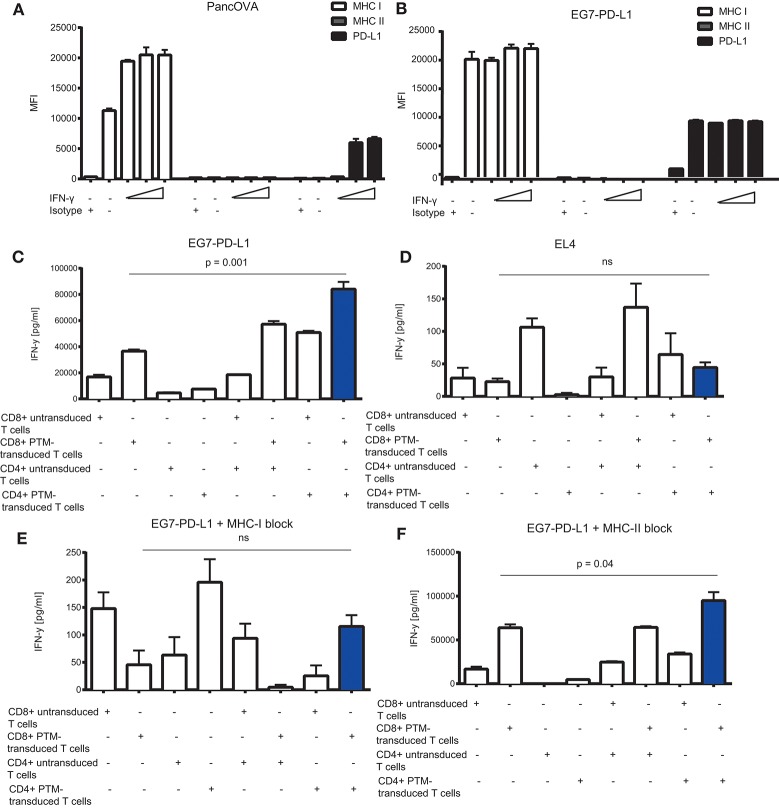
*In vitro* characterization of the MHC I, MHC II, and PD-L1 expression on tumor cells and its effect on interferon-γ (IFN-γ) release by PD1-CD28 fusion protein (PTM)- transduced CD4+ and CD8+ T cells in T cell-tumor cell cocultures. **(A)** Panc02-OVA or **(B)** E.G7-OVA-PD-L1 cells were stimulated with increasing concentrations of recombinant murine IFN-γ (2, 20, 100 ng/ml) during a 48 h period. MHC I, MHC II, and PD-L1 expression was assessed by flow cytometry. **(C–F)** PTM-transduced or untransduced primary murine OT-1, PTM-transduced or untransduced primary murine OT2 T cells or OT1 plus OT-2 T cells were prestimulated for 24 h with anti-CD3 antibody plus recombinant PD-L1. T cells were then cocultured with EL4 **(D)** or with E.G7-OVA-PDL-1 in the absence **(C)** or presence of neutralizing anti-MHC. I antibody **(E)** or of neutralizing anti-MHCII-antibody **(F)**. The resulting interferon-γ (IFN-γ) release was measured by enzyme-linked immunosorbent assay (ELISA). Experiments **(A–F)** were performed in triplicates. Experiments **(C–F)** are representative of two independent experiments. Bars represent SEM and *P* values from Student's *t*-test are shown. All tests are two-sided.

To further delineate the synergistic action of OT1-PTM and OT2-PTM T cells, we addressed the expression of potential components of the system on the tumor cell side. We therefore analyzed MHC I for OT1 T cell recognition, PD-L1 for PTM-T cell activation and MHC II for OT2 T cell activation. In both models—Panc02-OVA and EG7-PD-L1—we found strong expression of MHC I but not of MHC II (Figures 4A,B). Not surprisingly, PD-L1 was constitutively overexpressed on EG7-PD-L1 and could be induced on Panc02-OVA upon IFN-γ stimulation (Figures 4A,B). Functionally, the observed synergy on EG7-PD-L1 of OT1-PTM and OT2-PTM T cells (Figure 4C) was entirely abrogated on OVA negative EL4 T cells (Figure 4D). Importantly this was not due to lack of MHC I or PD-L1 expression (not shown). Identical results were found when EG7-PD-L1 were pretreated with MHC I-blocking antibodies. As in the absence of OVA, T cell activity was entirely abrogated (Figure 4E). In contrast, MHC II-blockade did not impact on T cell recognition by combined OT1-PTM and OT2-PTM T cells (Figure 4F). These results indicate that both PD-L1 and MHC I but not MHC II are essential for the activity of our proposed strategy.

## Discussion

ACT, especially for solid tumors, is often limited by the immunosuppressive tumor milieu. Tumor cells evade an efficient tumor immune response especially via the PD-1-PD-L1 axis. Here, we report that CD4+ T cells, expressing a PD1-CD28 fusion receptor, have the potential to overcome PD-L1-mediated T cell suppression. We hypothesized that PTM-transduced CD4+ T cells might further boost the efficacy of CD8+ T cells *in vitro*, pointing toward potential avenues for translation of the approach.

Inhibitory receptors, such as PD-1 and CTLA-4, are important checkpoint molecules that prevent autoimmunity under physiological conditions. However, when expressed by tumor-infiltrating T cells these molecules strongly prevent an effective anti-tumor response. Following a similar strategy, a costimulatory CTLA-4–CD28 fusion receptor was shown to induce large amounts of IL-2 and high proliferation of CD4+ T cells when introduced in the latter, strengthening the idea of such fusion proteins to support CD4+ T cell activity (38). We previously described a PD1-CD28 fusion protein that rendered antigen-specific CD8+ T cells resistant to PD-1-PD-L1-mediated anergy. Thus, we wondered if this would also apply to CD4+ T cells in a similar fashion (32). In the present manuscript, we could indeed transfer the activity of PTM to CD4+ T cells boosting T cell proliferation and cytokine production in the presence of cancer cells, which further underlines underpinning previous data using a CTLA-4-CD28 fusion receptor.

CD4+ T cells exert potent anti-tumoral effects on their own right (15, 10). This can be mediated either through direct recognition of MHC II+ tumor cells or indirectly through secretion of IFN-γ and activation of bystander myeloid cells (15). In addition, CD4+ T cells can support and contribute to CD8+ T cell function (39–42). If transferred adoptively, CD4+ T cells can even rescue anergic tumor infiltrating CD8+ T cells by T cell help (43). As our fusion protein essentially seems to further boost the function of the cell subsets either alone or in combination, we indeed observed that also the collaboration between CD4+ and CD8+ T cells was enhanced through introduction of PTM in both cell types. Interestingly, this effect was dependent on an optimal CD4+ to CD8+ T cell ratio, which is also in line with clinical observations observed with CAR T cells (7, 8). This is further confirmed in multiple studies dealing with mixtures of CD4+ and CD8+ T cells for ACT (16–18). Notwithstanding the role of PD-1-mediated anergy, we argue and show that this brake is released by our PD1-CD28 fusion protein. Similar observations were reported with CTLA-4-CD28-expressing CD4+ and CD8+ T cells (38). Mechanistically, IL-2 derived from CD4+ T cells seems to mediate the synergistic effect of PD1-CD28 fusion receptor-transduced CD4+ and CD8+ T cells. As IL-2 improves CD8+ T cell activation, proliferation, and persistence one could assume that the additional transfer of CD4+ T cells would allow a lower dose of CD8+ T cells per patient. This would come with the additional advantage, that systemic IL-2 administration which often accompanies ACT protocols and causes significant side effects, could be prevented (44, 45). CD4+ T cells are also important for long-term protective anti-tumoral immunity (46, 47). In our hands, transduced CD4+ and CD8+ T cells predominantly developed a central memory phenotype. At least for CD8+ T cells longer persistence of CD8+ clones isolated from central memory T cells as compared to clones from CD8+ effector cells was observed *in vivo* after T cell transfer. This further indicates the importance of specific T cell subset functions for effective adoptive immunotherapy (48). An open question remains how CD4+-T cells would sense their antigen *in vitro*. We could demonstrate that OVA expression by the tumor cells, MHC I presentation and recognition of MHC I presented peptide by cocultured CD8+ T cells was mandatory for CD4+ T cell action. CD4+ T cells in general and OT-2 T cells in particular can be stimulated MHC II independently in the presence of large amounts of soluble antigen (49). OVA is known to be secreted by cells stably transfected with it and additional antigen release by CD8+-OT-1-T cells might lead to the level of antigen required for CD4+ T cells *in vitro*. The exact role of this known mechanism *in vivo* is currently unclear but has been repeatedly shown in several models (15). In any case, the *in vivo* activity observed strongly suggests translational potential for this strategy. An open question is how much data from the OT-1-OT-2 system will be transferrable to endogeneous antigens and to TCRs with different affinities. This antigen system is one of the most widely tested systems in T cell research. A significant amount of our knowledge has been generated in these models. Several studies suggest that data gathered from such preclinical studies will actually translate to clinical studies, corroborating the value of the OT-1-OT-2 system for translational T cell research (50, 51).

Antibodies, such as nivolumab, targeting the PD-1-PD-L1 axis can revive exhausted CD8+ T cells and have demonstrated impressive clinical activity (52, 53). However, more than 50% of PD-L1-positive tumors do not respond to anti-PD-L1/PD-1 antibody treatment (54). In addition, treatment protocols using those antibodies often require multiple injections and cause significant toxicities to the patient (55). Based on our previous data we assume that a single dose of PD1-CD28 fusion receptor-transduced CD4+ and CD8+ T cells would induce tumor regression *in vivo*, significantly lowering potential side effects due to systemic T cell activation (32). Even PD-L1 negative tumors could be targeted by our combinatorial approach. Transduced CD4+ T cells can also be activated by interaction with PD-L2, another ligand of PD-1, expressed on antigen-presenting cells present in the tumor microenvironment.

In summary, our results indicate that PD1-CD28 fusion protein transduced CD4+ T cells have the potential to overcome the PD-1-PD-L1 immunosuppressive axis in pancreatic cancer and non-Hodgkin-lymphoma. Collectively, inhibiting PD-1 signaling in both CD4+ and CD8+ T cells might be the most effective way to enhance antitumor immunity. This data will need to be further investigated in other models while moving the approach toward translation.

## Author contributions

FR designed experiments, supervised experiments, discussed data, and wrote the manuscript. FK designed experiments, conducted experiments, analyzed data, and wrote the manuscript. MC, SG, CH, and BC conducted experiments. PD and SE wrote the manuscript; SK designed and conceptualized the research, supervised the experiments, discussed data, and wrote the manuscript.

### Conflict of interest statement

Data of this work have been generated for the doctoral thesis of FK and BC at the Ludwig-Maximilians-Universität München. The remaining authors declare that the research was conducted in the absence of any commercial or financial relationships that could be construed as a potential conflict of interest.

## Supplementary material

Supplementary Figure 1 Differentiation of PD1-CD28 fusion protein (PTM)-transduced CD4+ and CD8+ T cells in T cell-tumor cell cocultures. **(A–D)** PTM-transduced, untransduced primary murine OT-1, PTM-transduced, untransduced primary murine OT-2 T cells or OT-1 together with OT-2 T cells were prestimulated for 24 h with anti-CD3 antibody and recombinant PD-L1. T cells were then cocultured with Panc02-OVA-PD-L1. CCR7 and CD62L expression on T cells was analyzed prior to and after 36 h of coculture by flow cytometry. Experiments **(A–D)** are representative of three independent experiments each performed at least in duplicates. Bars represent SEM.

Supplementary Figure 2 Cytotoxic activity of PD1-CD28 fusion protein (PTM)-transduced CD4+ and CD8+ T cells in T cell-tumor cell coculture in the presence of an Interleukin-2 (IL-2) neutralizing antibody. PTM-transduced, untransduced primary murine OT-1, PTM-transduced, untransduced primary murine OT-2 T cells or OT-1 together with OT-2 T cells were prestimulated for 24 h with anti-CD3 antibody and recombinant PD-L1. T cells were then cocultured with Panc02-OVA in the presence or absence of neutralizing anti-IL-2 antibody and LDH release from lysed tumor cells was measured. The experiment was performed in quadruplicates. Bars represent SEM and *P* values from Student's *t*-test are shown. All tests are two-sided.
